# Intraoperative Hepatic Blood Inflow Can Predict Early Acute Kidney Injury following DCD Liver Transplantation: A Retrospective Observational Study

**DOI:** 10.1155/2019/4572130

**Published:** 2019-08-06

**Authors:** Ao Jiao, Qingpeng Liu, Feng Li, Rui Guo, Bowen Wang, Xianliang Lu, Ning Sun, Chengshuo Zhang, Xiaohang Li, Jialin Zhang

**Affiliations:** ^1^Hepatobiliary Surgery Department and Unit of Organ Transplantation, The First Hospital of China Medical University, Shenyang, China; ^2^Department of Hepatopancreatobiliary Surgery, Cancer Hospital of China Medical University, Liaoning Cancer Hospital & Institute, Shenyang, China

## Abstract

**Purpose:**

Acute kidney injury (AKI) is a major and severe complication following donation-after-circulatory-death (DCD) liver transplantation (LT) and is associated with increased postoperative morbidity and mortality. However, the risk factors and the prognosis factors of AKI still need to be further explored, and the relativity of intraoperative hepatic blood inflow (HBI) and AKI following LT has not been discussed yet. The purpose of this study was to investigate the correlation between HBI and AKI and to construct a prediction model of early acute kidney injury (EAKI) following DCD LT with the combination of HBI and other clinical parameters.

**Methods:**

Clinical data of 132 patients who underwent DCD liver transplantation at the first hospital of China Medical University from April 2005 to March 2017 were analyzed. Data of 105 patients (the first ten years of patients) were used to develop the prediction model. Then we assessed the clinical usefulness of the prediction models in the validation cohort (27 patients). EAKI according to Kidney Disease Improving Global Outcomes (KDIGO) criteria based on serum creatinine increase during 7-day of postoperative follow-up.

**Results:**

After Least Absolute Shrinkage and Selection Operator (LASSO) regression and simplification, a simplified prediction model consisting of the Child-Turcotte-Pugh (CTP) score (p=0.033), anhepatic phase (p=0.014), packed red blood cell (pRBC) transfusion (p=0.027), and the HBI indexed by height (HBI/h) (p=0.002) was established. The C-indexes of the model in the development and validation cohort were 0.823 [95% CI, 0.738-0.908] and 0.921 [95% CI, 0.816-1.000], respectively.

**Conclusions:**

In this study, we demonstrated the utility of HBI/h as a predictor for EAKI following DCD LT, as well as the clinical usefulness of the prediction model through the combination of the CTP score, anhepatic phase, pRBC transfusion and HBI/h.

## 1. Introduction

Acute kidney injury (AKI) is a major and severe complication following orthotopic liver transplantation and is associated with poor graft survival and increased mortality [1, 2]. The incidence of AKI after liver transplantation (LT) is high and ranges from 20 to 64% [1, 3–8].

Currently, there is no effective therapy or preventive strategy available for AKI after LT [9], although several promising strategies have been investigated [10–12]. Therefore, to predict the occurrence of AKI after LT and treat AKI as early as possible will significantly improve the outcome of liver transplantation.

Although a number of studies have evaluated AKI after LT, the clinical risk factors, especially intraoperative risk factors, still need to be further explored. Most of the studies reported risk factors of AKI after LT including longer anhepatic phase [13], larger red blood cell (RBC) transfusion amount [5, 14], and higher Child-Turcotte-Pugh (CTP) score [15]. In addition, some hemodynamic factors such as intraoperative mean arterial blood pressure [16], the use of intraoperative venovenous bypass technology [17], central venous pressure, right ventricular end-diastolic volume, and mixed venous oxygen saturation [6] have been reported to significantly influence the occurrence of postoperative AKI.

Twenty-five percent of the cardiac output flows through the liver, and hepatic blood inflow (HBI) changes will influence systemic hemodynamics [18]. However, whether intraoperative HBI including hepatic artery flow (HAF) and portal vein flow (PVF) following reconstruction has a correlation with AKI following LT is unknown.

Therefore, the aim of this study was to investigate the relationship between HBI and early acute kidney injury (EAKI) after DCD LT as well as develop and validate a prediction model for EAKI after DCD LT using preoperative and intraoperative factors including HBI.

## 2. Materials and Methods

### 2.1. Design and Patients

This retrospective observational study was approved by the institutional review board of our institution (the First Hospital of China Medical University). We retrospectively reviewed the electronic medical records of 156 consecutive adult patients who underwent DCD LT at our institution between April 2005 and March 2017. The need for informed consent was waived given the study's retrospective design. Patients without complete intraoperative hepatic blood flow data or postoperative serum creatinine monitoring data were excluded (n = 15). Patients with renal replacement therapy (RRT) were excluded (n = 1). Patients who underwent combined liver-kidney transplantation (n = 2) or died within 48 hours postoperatively (n = 5) were excluded. Patients with perirenal hematoma after LT (n=1) were excluded. The remaining 132 patients were analyzed. Then, 105 patients were selected from April 2005 to March 2015 to form the development cohort of this study. The remaining 27 patients from April 2015 to March 2017 were evaluated to form a validation cohort.

### 2.2. Definition of EAKI

AKI was defined according to the KDIGO criteria [19]: an increase in serum creatinine by 0.3 mg/dL ( ≥26.5 *μ*mol/L) within 48 hours or an increase in creatinine to ≥ 1.5 times baseline within the first 7 postoperative days, which have been validated in patients undergoing LT [6, 7, 20]. We determined postoperative early acute kidney injury (EAKI) according to the KDIGO criteria mentioned above during 7 days of postoperative follow-up. A urine output criterion was not used. All patients who met the KDIGO criteria within 7 days after LT were classified as having EAKI.

### 2.3. Intraoperative Hepatic Blood Flow Monitoring

Our center used Transonic HT313 Flowmeter (Ithaca, New York, USA) to measure the reconstructed HAF and PVF before abdominal closure. The HAF is the sum of the mean blood flow of reconstructed hepatic arteries. The PVF represents intraoperative mean portal vein flow. The HBI is the sum of HAF and PVF.

### 2.4. Statistical Analysis

#### 2.4.1. Patient Characteristics

The Shapiro test was used to determine the normality of the data. Either Fisher's exact test or *χ*^2^ test was used to compare the categorical variables between EAKI and non-EAKI patients. Comparisons of continuous variables between EAKI and non-EAKI patients were performed with Student's T tests or Mann-Whitney U tests.

#### 2.4.2. Prediction Model Building

The Least Absolute Shrinkage and Selection Operator (LASSO) regression [21] was used to select predictors and eliminate multicollinearity. Logistic regression was used to identify univariate and multivariate predictors for EAKI. Model A was developed in the development cohort by multivariable logistic regression whose variables were selected by LASSO regression based on the one standard error criteria [22]. Model B is a simplified model of model A.

#### 2.4.3. Models Evaluation

Calibration of the models was assessed using Hosmer-Lemeshow goodness-of-fit statistics. To evaluate the prediction performance of the models, the Harrell's concordance index (C-index) was measured in both the development and validation cohort. A calibration curve was plotted to compare the agreement between observed outcomes (Y-axis) and the predictions of the model (X-axis). Finally, to facilitate calculation of individualized risk in clinical practice, model B was converted to an easy-to-use nomogram.

#### 2.4.4. Software

R software (Version 3.5.1, http://www.r-project.org) was used for the statistical analysis and plot generation, with rms, foreign, pROC, psych, glmnet, and ResourceSelection packages. P < 0.05 was considered statistically significant.

## 3. Results

### 3.1. Patient Characteristics

Patient demographics and perioperative variables according to the diagnosis of EAKI are presented in Table 1. EAKI occurred in 31 patients (29.5%) in the development cohort. There were significant differences in liver tumor (p=0.027), preoperative total bilirubin level (p=0.003), preoperative MELD score (p=0.015), preoperative CTP score (p=0.003), cold ischemic time (CIT) (p=0.028), operation time (OT) (p=0.015), anhepatic phase (AP) (p=0.002), packed red blood cell (pRBC) transfusion (p=0.005), cryoprecipitate transfusion (p=0.035), PVF (p=0.015), and HBI (p=0.015) between EAKI and non-EAKI patients in the development cohort. However, for HAF, there was no significant difference (p=0.905) between EAKI and non-EAKI patients.

### 3.2. Prediction Model Building

**Figure 1:**
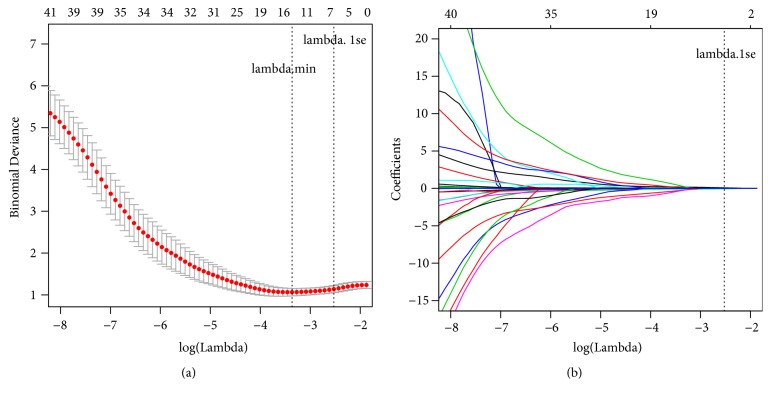
EAKI predictors screened in the development cohort using LASSO regression. (a) Selection of tuning parameter (*λ*) in the LASSO regression via 10-fold cross-validation in the development cohort. Binomial deviances from the LASSO regression's cross-validation procedure were plotted as a function of log(*λ*). *λ* is the tuning parameter. Y-axis indicates binomial deviances. The lower x-axis indicates log(*λ*). Numbers along the upper x-axis represent the average number of predictors. Red dots indicate average deviance values for each model with given *λ*, and vertical bars through the red dots show the upper and lower values of the deviances. The vertical black lines define the optimal values of *λ*, where the model provides its best fits to the data. Lambda.min corresponds to the *λ* which minimizes mean squared error and was used for variable selection. Lambda.1se corresponds to the *λ* that is one standard error from the lambda.min. (b) LASSO coefficients produced by the regression analysis (in (a)). A vertical line at x-axis with log (*λ*) = -2.434 was generated based on the one standard error criteria in 10-fold cross-validation procedure. The 6 resulting predictors with nonzero coefficients were indicated in the plot.

As shown in Figures 1(a) and 1(b), all continuous and categorical variables in the development cohort were performed by LASSO regression analysis using 10-fold cross-validation and the one standard error criterion. When parameter *λ* corresponds to the minimum-deviance within one standard error, 6 variables including CTP score, CIT, AP, HBI/h, OT, and pRBC transfusion were obtained. Then, the 6 variables were selected to build model A using multivariable logistic regression. Model B, a simplified model of model A, was built via eliminating CIT (p=0.211) and OT (p=0.212) from model A (Table 2). After the Hosmer-Lemeshow test, model A and model B were a good fit for the data (Table 3). The two prediction models can be shown in the following equations. 

*Model A*

(1) $$\begin{array}{c} P_{EAKI}=1-\frac{1}{e^{-6.9+0.29\times CTPscore+0.3\times CIT/100+0.16\times AP/10-0.28\times HBI/h+0.37\times OT/100+0.19\times pRBC/1000}} \end{array}$$

*Model B*

(2) PEAKI=1−1e−4.42+0.26×CTPscore+0.27×AP/10−0.26×HBI/h+0.21×pRBC/1000

P_EAKI_: probability of postoperative EAKI occurrence.

### 3.3. Prediction Model Performance Assessment

**Figure 2:**
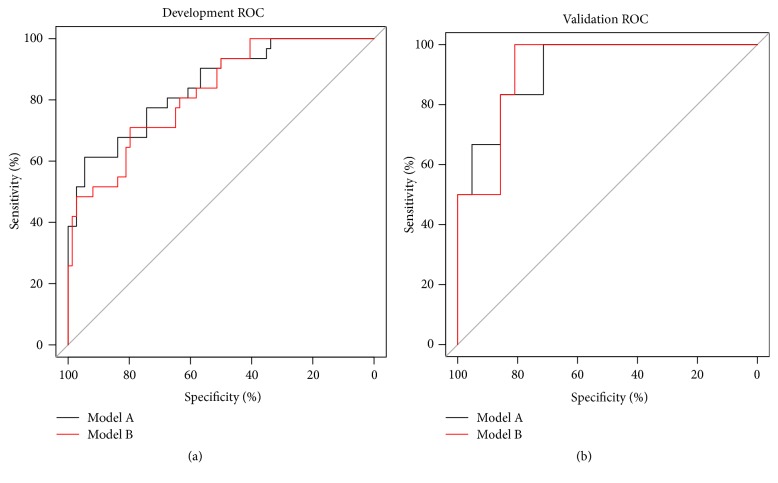
ROC curves for prediction of EAKI by the models. (a) Development cohort. The C-index of each model is 0.847 [95% CI, 0.765-0.928] in model A; 0.823 [95% CI, 0.738-0.908] in model B. (b) Validation cohort. The C-index of each model is 0.921 [95% CI, 0.809-1.000] in model A; 0.921 [95% CI, 0.816-1.000] in model B.

**Figure 3:**
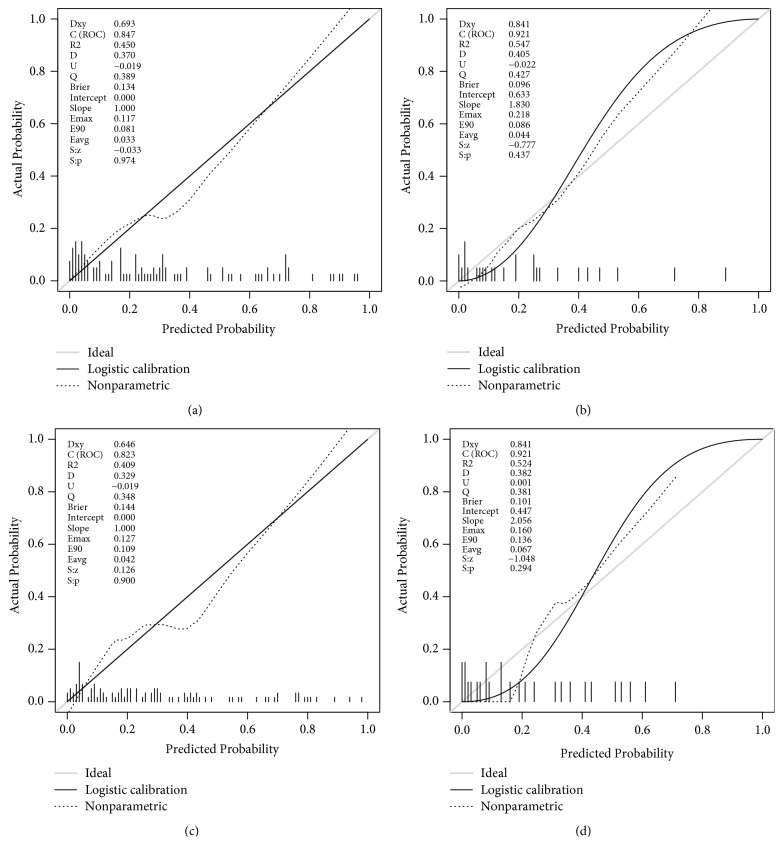
Calibration curves of models. (a-d) A calibration curve was plotted to compare the agreement between observed outcomes (Y-axis) and the predictions of the model (X-axis). (a) Calibration curves of model A in the development cohort. (b) Calibration curves of model A in the validation cohort. (c) Calibration curves of model B in the development cohort. (d) Calibration curves of model B in the validation cohort.

**Figure 4:**
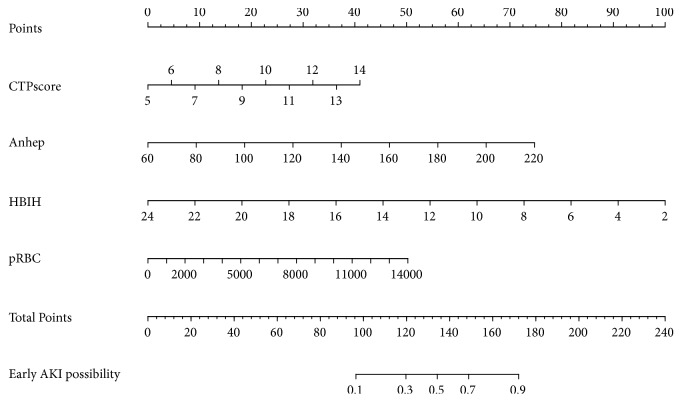
The nomogram of model B for predicting incidence of EAKI following DCD LT. CTP score, Child-Turcotte-Pugh score; AP, anhepatic phase, min; pRBC, packed red blood cells transfusion, ml; HBI/h, hepatic blood inflow indexed by height, ml/min/cm. Draw an upward vertical line to the “Points” bar to calculate points. Based on the sum, draw a downward vertical line from the “Total Points” line to calculate EAKI possibility after DCD LT.

The receiver operating characteristic (ROC) curves of model A and model B in the development and validation cohort were shown in Figure 2. In the development cohort, the C-indexes of model A and B were 0.847 [95% confidence interval (CI), 0.765-0.928] and 0.823 [95% CI, 0.738-0.908], respectively, and there was no significant difference (p = 0.167, DeLong's test) in the C-indexes between the two models. In the validation cohort, the C-indexes of model A and B were 0.921 [95% CI, 0.809-1.000] and 0.921 [95% CI, 0.816-1.000], respectively, and there was no significant difference (p = 1.000, DeLong's test) in the C-indexes between the two models (Table 4). The calibration curves of models were shown in Figure 3, and slopes of mode A in the development cohort and validation cohort were 1.000 and 1.830, respectively. Slopes of mode B in the development cohort and validation cohort were 1.000 and 2.056. Finally, the nomograms of model B were shown in Figure 4.

## 4. Discussion

In this retrospective observational study, we made two important clinical findings. First, the HBI/h was a key risk factor in EAKI occurrence after DCD LT. Second, the prediction models combining with the HBI/h and other clinical parameters could accurately predict EAKI occurrence after DCD LT and had clinical value.

A previous study has reported that predictors for postoperative late acute renal failure (ARF) differ from early ARF and correspond to postoperative parameters [23]. Therefore, we built prediction models using preoperative and intraoperative parameters to predict postoperative early AKI.

AKI after LT is multifactorial in origin. Higher CTP score [15], longer cold ischemic time[24], longer anhepatic phase [13], longer operation time [1], and larger pRBC transfusion amount [5, 14] have been reported as high-risk factors or predictors of AKI after living-donor or cadaveric LT.

Higher MELD scores or CTP scores suggest worse pretransplant liver reservation function. And a higher MELD score may suggest worse pretransplant kidney function, either. Many studies have shown an association between preoperative MELD score and postoperative AKI [1, 4, 13]. In our study, MELD score was eliminated and CTP score was selected by LASSO regression; this might because there was no significant difference in baseline renal function between EAKI and non-EAKI patients.

Hepatic ischemia-reperfusion injury (HIRI) plays a critical role in the pathogenesis of AKI after LT [25]. HIRI is associated with a systemic inflammatory response, which may cause AKI through hemodynamic mechanisms and direct tubular cell death [26]. Thus, long warm ischemic time (WIT) and CIT will increase the probability of postoperative AKI theoretically. WIT had been considered as a key predictor of AKI following LT [7, 8]. But there was no significant difference (p=0.623) in WIT between EAKI and non-EAKI patients because WIT was strictly controlled in our center. CIT as a risk factor of AKI after LT remains controversial [7, 24]; this may be because different donor criteria or different cold storage conditions were used in different centers. In our study, there was no significant difference (p=0.883) in CIT between EAKI and non-EAKI patients in the validation cohort. So CIT as a predictor of EAKI needs to be reevaluated using a larger sample size in the future.

Systemic hemodynamic changes after LT are the cause of postoperative AKI [27]. Longer anhepatic phase, longer operation time, and larger pRBC transfusion all influence systemic hemodynamics and then influence renal blood perfusion. HBI accounts for about 25% of cardiac output [18] and HBI will change more or less after blood vessel reconstruction in LT. Our study suggested EAKI was relevant to HBI and PVF. Interestingly, after eliminating multicollinearity in LASSO regression, HBI/h was selected into prediction models, but how HBI influence renal blood flow needs to be investigated in future research.

Studies have shown that the incidence of ARF after classic orthotopic LT is significantly higher than that of ameliorative piggyback liver transplantation (APBLT) [27]. In this study, APBLT was performed in DCD LT.

This study has limitations. First, although our prediction model satisfies the minimum sample size requirement [28], the larger sample size is needed to verify the accuracy of the prediction model. Second, the models should be validated prospectively at other centers to demonstrate its applicability, however, there is no HBI data from other centers could be available in present. Third, during LT, we did not directly monitor cardiac output and kidney flow which would reflect perfusion of kidney and predict postoperative EAKI better. In addition, severe stages of EAKI were not analyzed because of the small sample size.

## 5. Conclusion

In this study, we demonstrated that HBI can be a reliable predictor of EAKI after DCD LT for the first time. In addition, we developed an easy and accurate prediction model including CTP score, AP, HBI/h and pRBC transfusion for EAKI after DCD LT.

## Figures and Tables

**Table 1:** Patients characteristics and perioperative parameters by KDIGO classification.

*Characteristics*	*Development cohort (n=105)*			*Validation cohort (n=27)*		
	non-EAKI (n=74)	EAKI (n=31)	*P *value	non-EAKI (n=21)	EAKI (n=6)	*P* value
*Demographic data*						
Age, years	50 [43-57]	53 [48-58]	0.066	48 [42-53]	52 [45-60]	0.458
Female, n	17 (23.0)	7 (22.6)	0.965	6 (28.6)	2 (33.3)	1.000
Height, cm	170 [165-175]	170 [165-175]	0.816	172 [170-176]	173 [163-177]	0.716
Weight, kg	65 [58-77]	65 [60-75]	0.737	68 [62-74]	66 [63-69]	0.533
BMI, kg/m^2^	22.9 [20.4-26.1]	22.2 [21.7-24.4]	0.645	22.3 [21.5-23.8]	23.3 [22.0-24.3]	0.971
BSA, m^2^	1.76 [1.63-1.91]	1.77 [1.65-1.91]	0.778	1.81 [1.72-1.92]	1.81 [1.69-1.84]	0.619
*Background medical status*						
Hypertension, n	4 (5.4)	2 (6.5)	0.802	0 (0.0)	1 (16.7)	0.222
Diabetes mellitus, n	9 (12.2)	6 (19.4)	0.512	4 (19.0)	2 (33.3)	0.588
Smoking, n	16 (21.6)	9 (29.0)	0.416	6 (28.6)	2 (33.3)	1.000
Alcoholic liver cirrhosis, n	5 (6.8)	1 (3.2)	0.802	1 (4.8)	1 (16.7)	0.402
HBV hepatitis, n	52 (70.3)	22 (71.0)	0.943	12 (57.1)	4 (66.6)	1.000
HCV hepatitis, n	7 (9.5)	2 (6.5)	0.904	2 (9.5)	1 (16.7)	0.545
Liver tumor, n	31 (41.9)	6 (19.4)	0.027	6 (28.6)	0 (0.0)	0.284
Cholestatic disease, n	6 (8.1)	5 (16.1)	0.382	2 (9.5)	0 (0.0)	1.000
Hepatic encephalopathy, n	12 (16.2)	10 (32.3)	0.065	2 (9.5)	1 (16.7)	0.545
Serum albumin level, g/L	31.5 [27.5-36.0]	30.0 [25.5-32.4]	0.122	36.1 [31.5-38.1]	32.7 [27.8-37.8]	0.585
Total bilirubin, *μ*mol/L	33.7 [20.1-75.5]	78.1 [39.3-273.9]	0.003	39.7 [21.9-99.7]	136.3 [103.5-191.4]	0.031
Prothrombin time, s	18.6 [15.7-25.1]	21.4 [18.3-25.7]	0.152	17.0 [15.3-19.4]	25.1 [22.3-28.3]	0.010
MELD score	12.6 [9.1-20.9]	17.8 [12.6-21.8]	0.015	13.2 [9.4-18.6]	21.5 [20.4-24.9]	0.031
CTP score	9 [7-11]	10 [10-11]	0.003	8 [6-9]	10 [10-11]	0.012
Child class, n (A/ B/ C)	16/24/34	1/8/22	0.020	7/9/5	0/0/6	0.003
*Baseline renal function*						
Preoperative serum creatinin, *μ*mol/L	62.0 [52.3-74.8]	65.0 [54.0-78.5]	0.421	61.0 [53.0-95.0]	56.0 [51.3-57.8]	0.122
Preoperative estimated	129.6	118.5	0.369	108.8	154.6	0.092
GFR, ml/min/1.73 m^2^	[105.0-161.7]	[98.9-147.7]		[78.4-152.6]	[139.7-172.7]	
Preoperative blood urea	5.20	4.80	0.464	5.94	3.75	0.216
nitrogen, mmol/L	[4.00-7.17]	[4.10-6.29]		[4.83-6.50]	[3.01-5.66]	
*Donor liver factors*						
Warm ischemic time, s	180 [150-180]	180 [150-195]	0.623	270 [180-300]	300 [210-300]	0.630
Cold ischemic time, min	493 [413-554]	540 [445-647]	0.028	440 [385-510]	418 [385-540]	0.883
*Operation and anesthesia details*						
Operation time, min	533 [480-600]	590 [530-678]	0.015	540 [480-600]	570 [518-690]	0.357
Anhepatic phase, min	103 [90-116]	112 [107-136]	0.002	80 [75-92]	95 [92-103]	0.152
Norepinephrine use, n	6 (8.1)	4 (12.9)	0.690	1 (4.8)	0 (0.0)	1.000
pRBC transfusion, mL	2800 [1275-4400]	4800 [2200-6450]	0.005	3200 [2000-4800]	4800 [2650-7400]	0.255
FFP transfusion, mL	1000 [600-1950]	1400 [900-2700]	0.066	1200 [550-2000]	2650 [1150-4000]	0.129
Platelet transfusion, units	0 [0-0]	0 [0-0]	0.357	0 [0-1]	0 [0-2]	0.857
Cryoprecipitate transfusion, units	0 [0-10]	10 [0-10]	0.035	0 [0-20]	15 [3-20]	0.378
PVF, mL/min	1730 [1385-2237]	1444 [1068-1898]	0.015	1670 [1200-2400]	1122 [1023-1183]	0.036
PVF/height, mL/min/cm	10.12 [8.35-13.22]	8.11 [6.33-10.76]	0.012	9.82 [7.26-13.34]	6.575 [6.06-7.04]	0.026
PVF/weight, mL/min/kg	27.59 [20.26-34.92]	20.23 [17.45-28.50]	0.013	26.94 [20.40-33.75]	16.86 [15.48-18.16]	0.026
PVF/BMI, m^2^·mL/min/kg	76.72 [58.29-104.11]	61.94 [48.33-77.44]	0.018	77.86 [58.54-103.36]	47.66 [42.70-53.52]	0.036
PVF/BSA, mL/min/m^2^	1000.0 [779.2-1243.4]	788.0 [643.1-1029.2]	0.009	970.9 [734.3-1279.9]	627.7 [588.4-681.2]	0.015
HAF, mL/min	149.0 [106.8-214.5]	165.0 [106.2-215.5]	0.905	226.0 [142.0-280.0]	167.0 [113.4-214.6]	0.431
HAF/height, mL/min/cm	0.86 [0.62-1.24]	0.98 [0.61-1.23]	0.905	1.28 [0.81-1.60]	0.94 [0.70-1.20]	0.254
HAF/weight, mL/min/kg	2.20 [1.63-3.22]	2.49 [1.47-3.12]	0.886	3.21 [2.26-4.31]	2.47 [1.76-3.09]	0.408
HAF/BMI, m^2^·mL/min/kg	6.38 [4.50-8.88]	7.00 [4.07-9.62]	0.855	9.39 [5.96-12.74]	7.57 [4.67-10.13]	0.196
HAF/BSA, mL/min/m^2^	83.38 [58.27-122.63]	96.13 [54.88-116.29]	0.902	134.64 [74.47-153.37]	91.16 [66.86-114.27]	0.260
HBI, mL/min	1899 [1570-2457]	1656 [1215-2082]	0.015	2170 [1480-2575]	1260 [1224-1318]	0.042
HBI/height, mL/min/cm	11.32 [9.43-14.32]	9.30 [7.23-11.99]	0.010	12.76 [9.22-14.97]	7.62 [7.39-7.80]	0.036
HBI/weight, mL/min/kg	30.05 [22.00-38.91]	24.01 [19.33-31.79]	0.014	29.63 [23.77-36.68]	19.61 [18.71-20.20]	0.049
HBI/BMI, m^2^·mL/min/kg	86.84 [63.64-111.89]	68.71 [51.98-88.65]	0.014	94.36 [68.69-111.31]	54.67 [49.78-58.68]	0.044
HBI/BSA, mL/min/m^2^	1098.6 [895.2-1377.7]	850.0 [702.8-1158.1]	0.006	1207.3 [858.9-1418.1]	731.1 [705.9-762.8]	0.036
Urine volume, mL	1300 [800-2000]	1300 [725-1725]	0.621	1300 [750-2000]	1000 [650-1180]	0.515
*Postoperative factors*						
Tacrolimus use, n	71 (95.9)	31 (100.0)	0.553	20 (95.2)	6 (100.0)	1.000
Cyclosporine use, n	3 (4.1)	0 (0.0)	0.553	1 (4.8)	0 (0.0)	1.000

BMI = body-mass index; BSA = body surface area; MELD = model for end-stage liver disease; CTP = Child-Turcotte-Pugh; GFR = glomerular filtration rate; pRBC = packed red blood cells; FFP = fresh frozen plasma; PVF = intraoperative mean portal vein flow; HAF = intraoperative mean hepatic artery flow; HBI = intraoperative mean hepatic blood inflow; Warm ischemic time: from no heartbeat to cold perfusion; Cold ischemic time: from cold perfusion to liver blood supply restored.

**Table 2:** Prediction model.

	Variables from LASSO regression (lambda.1se)			Model A (LASSO variables)			Model B (Simplified)		
*Variable*	Coef	95%CI	p-value	Coef	95%Cl	p-value	Coef	95%Cl	p-value
CTP score	0.32	(0.12, 0.55)	0.003	0.29	0.05, 0.56	0.022	0.26	(0.03, 0.52)	0.033
CIT / 100	0.45	(0.10, 0.83)	0.015	0.30	-0.16, 0.78	0.211			
AP /10	0.30	(0.12, 0.50)	0.002	0.16	-0.07, 0.41	0.185	0.27	(0.07, 0.50)	0.014
HBI / h	-0.17	(-0.30, -0.05)	0.008	-0.28	-0.47,-0.13	0.001	-0.26	(-0.45, -0.11)	0.002
OT / 100	0.58	(0.19, 1.01)	0.005	0.37	-0.18, 0.99	0.212			
pRBC /1000	0.20	(0.07, 0.35)	0.005	0.19	0.01, 0.39	0.051	0.21	(0.03, 0.40)	0.027
*AIC*					101.54			101.84	

**Table 3:** Hosmer and Lemeshow goodness of fit (GOF) test.

*Model*	*Hosmer and Lemeshow goodness of fit (GOF) test*		
	X-squared	df	p-value
Model A	8.068	8	0.427
Model B	5.473	8	0.706

**Table 4:** The C-index of the models.

Model	Development cohort	Validation cohort
	C-index (95%CI)	C-index (95%CI)
Model A	0.847 (0.765-0.928)	0.921 (0.809-1.000)
Model B	0.823 (0.738-0.908)	0.921 (0.816-1.000)
Comparison of AUC (p-value)	0.167	1.000

## Data Availability

The patients' data used to support the findings of this study are included within the supplementary information file.

## Supplementary Materials

Supplementary Materials The csv file is the anonymous data set of patients.

## References

[1] Park M. H., Shim H. S., Kim W. H. (2015). Clinical risk scoring models for prediction of acute kidney injury after living donor liver transplantation: a retrospective observational study. *PLoS ONE*.

[2] Levitsky J., O'Leary J. G., Asrani S. (2016). Protecting the kidney in liver transplant recipients: practice-based recommendations from the american society of transplantation liver and intestine community of practice. *American Journal of Transplantation*.

[3] Umbro I., Tinti F., Mordenti M. (2011). Model for end-stage liver disease score versus simplified acute physiology score criteria in acute renal failure after liver transplantation. *Transplantation Proceedings*.

[4] O'Riordan A., Wong V., McQuillan R., McCormick P. A., Hegarty J. E., Watson A. J. (2007). Acute renal disease, as defined by the rifle criteria, post-liver transplantation. *American Journal of Transplantation*.

[5] Chen J., Singhapricha T., Hu K. (2011). Postliver transplant acute renal injury and failure by the rifle criteria in patients with normal pretransplant serum creatinine concentrations: a matched study. *Transplantation*.

[6] Kim W. H., Oh H., Yang S. (2019). Intraoperative hemodynamic parameters and acute kidney injury after living donor liver transplantation. *Transplantation*.

[7] Kalisvaart M., Schlegel A., Umbro I. (2018). The impact of combined warm ischemia time on development of acute kidney injury in donation after circulatory death liver transplantation. *Transplantation*.

[8] Kalisvaart M., Schlegel A., Umbro I. (2019). The AKI Prediction Score: a new prediction model for acute kidney injury after liver transplantation. *HPB*.

[9] Durand F., Francoz C., Asrani S. K. (2018). Acute kidney injury after liver transplantation. *Transplantation*.

[10] Burns K. E., Chu M. W., Novick R. J. (2005). Perioperative N-acetylcysteine to prevent renal dysfunction in high-risk patients undergoing cabg surgery. *Journal of the American Medical Association*.

[11] Kim W. H., Lee J.-H., Ko J. S. (2014). Effect of remote ischemic postconditioning on patients undergoing living donor liver transplantation. *Liver Transplantation*.

[12] Gassanov N., Nia A. M., Caglayan E., Er F. (2014). Remote ischemic preconditioning and renoprotection: from myth to a novel therapeutic option?. *Journal of the American Society of Nephrology*.

[13] Kundakci A., Pirat A., Komurcu O. (2010). Rifle criteria for acute kidney dysfunction following liver transplantation: incidence and risk factors. *Transplantation Proceedings*.

[14] Gallardo M. L., Herrera Gutierrez M. E., Pérez G. S., Balsera E. C., Fernández Ortega J. F., García G. Q. (2004). Risk factors for renal dysfunction in the postoperative course of liver transplant. *Liver Transplantation*.

[15] Hilmi I., Damian D., Al-Khafaji A. (2015). Acute kidney injury following orthotopic liver transplantation: incidence, risk factors, and effects on patient and graft outcomes. *British Journal of Anaesthesia*.

[16] Rueggeberg A., Boehm S., Napieralski F. (2008). Development of a risk stratification model for predicting acute renal failure in orthotopic liver transplantation recipients. *Anaesthesia*.

[17] Sun K., Hong F., Wang Y. (2017). Venovenous bypass is associated with a lower incidence of acute kidney injury after liver transplantation in patients with compromised pretransplant renal function. *Anesthesia & Analgesia*.

[18] Lautt W. W. (2009). *Hepatic Circulation: Physiology And Pathophysiology*.

[19] Kellum J. A., Lameire N., Aspelin P. (2012). Kidney disease: improving global outcomes (KDIGO) acute kidney injury work group. KDIGO clinical practice guideline for acute kidney injury. *Kidney International Supplements*.

[20] Leithead J. A., Armstrong M. J., Corbett C. (2014). Split liver transplant recipients do not have an increased frequency of acute kidney injury. *Transplant International*.

[21] Tibshirani R. (1996). Regression shrinkage and selection via the lasso. *Journal of the Royal Statistical Society: Series B (Statistical Methodology)*.

[22] Tibshirani R., Walther G., Hastie T. (2001). Estimating the number of clusters in a data set via the gap statistic. *Journal of the Royal Statistical Society B: Statistical Methodology*.

[23] Cabezuelo J. B., Ramírez P., Ríos A. (2006). Risk factors of acute renal failure after liver transplantation. *Kidney International*.

[24] Khosravi M. B., Milani S., Kakaei F. (2013). Serum neutrophil gelatinase-associated lipocalin versus serum creatinine for the prediction of acute kidney injury after liver transplantation. *International Journal of Organ Transplantation Medicine*.

[25] Leithead J. A., Tariciotti L., Gunson B. (2012). Donation after cardiac death liver transplant recipients have an increased frequency of acute kidney injury. *American Journal of Transplantation*.

[26] Leithead J. A., Armstrong M. J., Corbett C. (2013). Hepatic ischemia reperfusion injury is associated with acute kidney injury following donation after brain death liver transplantation. *Transplant International*.

[27] Brescia M. D., Massarollo P. C., Imakuma E. S., Mies S., Herrero J. I. (2015). Prospective randomized trial comparing hepatic venous outflow and renal function after conventional versus piggyback liver transplantation. *PLoS ONE*.

[28] Concato J., Feinstein A. R., Holford T. R. (1993). The risk of determining risk with multivariable models. *Annals of Internal Medicine*.

